# Is Lipoprotein(a) the Most Important Predictor of Residual Atherosclerotic Cardiovascular Disease Risk?

**DOI:** 10.3390/jcm11154380

**Published:** 2022-07-28

**Authors:** Nathan D. Wong

**Affiliations:** Heart Disease Prevention Program, Division of Cardiology, University of California, Irvine, CA 92697, USA; ndwong@hs.uci.edu

Lipoprotein(a) is an underrecognized, but significant genetic risk factor for atherosclerotic cardiovascular disease (ASCVD), shown to be causal from data from prospective epidemiologic studies, Mendelian randomization, and genome wide association studies [1]. With therapies in development targeting reduction of lipoprotein(a), it is important to better understand the strength of its prediction, especially in relation to other lipid and non-lipid determinants of cardiovascular outcomes.

What is not clear is its relative importance to other prognostic factors in persons with ASCVD. Moreover, not well-described is the relative importance of lipoprotein(a) once LDL-C is well-controlled in statin-treated ASCVD patients, given that this is the current standard of care in such patients. Most studies have not quantitatively reported on the relative contribution of lipoprotein(a) with other risk factors for the prediction of ASCVD events, even though such analyses are available. We examined this issue in a secondary analysis of the previously reported “Atherothrombosis Intervention in Metabolic Syndrome with Low HDL/High Triglycerides: Impact on Global Health Outcomes (AIM-HIGH)” clinical trial [2]. This trial studied 3414 participants aged 45 years or older with documented ASCVD including either coronary artery disease, cerebrovascular or carotid disease, or symptomatic peripheral arterial disease in addition to having atherogenic dyslipidemia defined as: (1) low density lipoprotein-cholesterol (LDL-C) of less than or equal to 160 mg/dL (4.1 mmol/L); (2) high density lipoprotein-cholesterol (HDL-C) of less than or equal to 40 mg/dL (1.0 mmol/L) for men or less than or equal to 50 mg/dL (1.3 mmol/L) for women; and (3) triglycerides greater than or equal to 150 mg/dL (1.7 mmol/L) and less than or equal to 400 mg/dL (4.5 mmol/L). Subjects were on statin therapy (40 mg simvastatin) and randomized to niacin versus placebo. The trial terminated early (at a mean follow-up of 3 years) due to a lack of efficacy for niacin in reducing ASCVD risk.

In our analysis of 3271 subjects with complete risk factor information, we created a 5-year risk prediction model (validated by 10-fold cross validation) for recurrent ASCVD events incorporating key variables of interest for the prediction of subsequent ASCVD events. We had follow-up for ASCVD events up to 6 years (mean 4.2 years), during which 16% of patients suffered a recurrent ASCVD event [3]. Our modelling considered key variables known to be associated with CVD and were available at baseline in our study, including age, sex, race, body mass index, blood pressure, LDL-C, HDL-C, triglycerides, lipoprotein(a), apolipoprotein A1, apolipoprotein B, smoking status, alcohol consumption, family history of cardiovascular disease (CVD), glycated hemoglobin, atrial fibrillation, serum creatine, homocysteine, specific ASCVD conditions (previous myocardial infarction, stroke, heart failure, carotid, or peripheral arterial disease), antihypertensive or diabetes drugs, aspirin use, previous use of higher versus lower intensity statins, body mass index, non-HDL-C, estimated glomerular filtration rate, pulse pressure, and treatment assignment. We allowed variables to enter if they were *p* < 0.15 in significance. *In the final prediction model, based on Wald Chi-square values, lipoprotein(a) was the strongest predictor of recurrent ASCVD events (Chi-sq = 18.2, p < 0.0001)*, followed by family history of cardiovascular disease (Chi-sq = 10.7, *p* = 0.001), homocysteine (Chi-sq = 9.5, *p* = 0.002), alcohol use (inversely) (Chi-sq = 6.0, *p* = 0.014), diabetes (Chi-sq = 5.9, *p* = 0.015), and male sex (Chi-sq = 5.0, *p* = 0.025). In our study, a 1 SD of lipoprotein(a) increment (37 nmol/L, or approximately 15 mg/dL) was associated with a 7% increase of ASCVD risk among our cohort of statin-treated patients with prior ASCVD. Of interest, neither age nor LDL-C entered the multivariable model; given that subjects were on statin therapy and LDL-C was well-controlled in many participants, the more limited range in LDL-C may have precluded its entry into the model. It is also possible other variables that were not available to us such as time since prior CVD event or number or severity of prior events could also have been important predictors of residual risk. To the best of our knowledge, our report is unique in quantifying lipoprotein(a) as the strongest predictor of ASCVD events, specifically in a secondary prevention population with known ASCVD on statin therapy.

As our study involved persons with ASCVD on statin therapy, it is possible that lipoprotein(a) is a stronger predictor of ASCVD in persons on statin therapy compared to not being on statin therapy. In a previously published meta-analysis of seven statin trials, Willeit et al. [4] showed lipoprotein(a) to predict future ASCVD event more strongly in those on statin treatment as compared to on placebo, with multivariable adjusted HR’s for those with lipoprotein(a) ≥50 mg/dL vs. <50 mg/dL of 1.47 and 1.26, respectively (*p* = 0.03 for interaction). Moreover, in our subsequent report from the AIM-HIGH trial [5] we showed among statin-treated patients with ASCVD that compared to lipoprotein(a) <15 mg/dL, those with levels of ≥70 mg/dL had an adjusted HR for first recurrent events of 1.77 and total recurrent events of 1.51 (both *p* < 0.0001). Also, is lipoprotein(a) a stronger predictor in primary or secondary prevention? While our study included only persons with known ASCVD, in the UK Biobank [6] a lipoprotein(a) of ≥150 nmol/L was present in 12.2% of those without and 20.3% of those with pre-existing ASCVD and was associated with HR, 1.50 (95% CI, 1.44–1.56) and HR, 1.16 (95% CI, 1.05–1.27) for incident ASCVD, respectively, showing lipoprotein(a) to be a stronger risk factor in primary prevention. However, in this study lipoprotein(a) was a weaker predictor for those on statins compared to those not on statins (interaction *p* < 0.0001). In a more recent report among 413,734, participants from UK Biobank [7], adding Lp(a) to a prediction model containing traditional CVD risk factors in the primary prevention group improved the C-index by 0.0017 (95% CI 0.0008–0.0026) and population attributable fractions (PAF) in the whole cohort of 5.8% and 3.0% were associated with Lp(a) values above 100 nmol/L and above 175 nmol/L, respectively. Moreover, in a meta-analysis of 17 studies including 283,328 patients specifically with known coronary disease, elevated lipoprotein(a) level was independently associated with the future risk of cardiac events (RR 1.78; 95% CI 1.31–2.42) as well as overall cardiovascular events (RR 1.29; 95% CI 1.17–1.42) [8]. A smaller study of only 258 patients [9] who had severe carotid and/or lower extremity disease, a lipoprotein(a) level >30 mg/dL did appear to be among the strongest predictors of the primary and secondary composite cardiovascular disease endpoints in multivariable analysis, with only prior myocardial infarction and/or ischemic stroke (and not age, sex, or other risk factors) also predicting outcomes. However, these studies did not rank the order of importance of variables in the prediction of future cardiovascular outcomes. In a much older study of examining the relative strength of predictors of angiographically-defined coronary artery disease, age, family history, apolipoprotein B, and lipoprotein(a) (in that order) were identified to be the most important predictors of coronary disease [10].

While our analysis from the AIM-HIGH cohort is a selected clinical trial sample, it provides evidence, at least in persons with known ASCVD on statin therapy, that lipoprotein(a) may be among the most important predictors of future cardiovascular disease outcomes. Future studies should report the relative contribution of lipoprotein(a) with other predictors for cardiovascular disease outcomes both among primary and secondary prevention cohorts and according to use of statin therapy. A better understanding of the relative strength of lipoprotein(a) in relation to other risk factors for predicting ASCVD events can serve to help inform future efforts to improve screening for and potentially treating elevated lipoprotein(a).
